# Senior Entrepreneurship: The Unrevealed Driver for Social Innovation

**DOI:** 10.3389/fsoc.2019.00030

**Published:** 2019-04-24

**Authors:** Justyna Stypińska, Annette Franke, Janina Myrczik

**Affiliations:** ^1^Department of Sociology, Institute for East European Studies, Free University Berlin, Berlin, Germany; ^2^Department for Social Work, University of Applied Sciences Ludwigsburg, Ludwigsburg, Germany

**Keywords:** senior entrepreneurship, aging, social innovation, business incubators, self-employment

## Abstract

From a political and economic perspective, senior entrepreneurship seems to be the response to the demographic consequences of the aging workforce in Europe. Several policies and strategies by the European Union (EU) and the Organization for Economic Co-operation and Development (OECD) promote senior entrepreneurship by creating a favorable environment and frameworks. This article examines the role of senior entrepreneurship as a social innovation understood as a response to unmet needs of population aging in the area of economic activity. In this paper draws on qualitative interviews with 6 experts and 4 senior entrepreneurs (as part of a larger research project) in Poland in addition to the analysis of reports and evaluations of incubator projects. Findings highlight the importance of other factors than financial sustainability of senior entrepreneurship: (1) social connectedness as a means against social isolation, (2) personal self-confidence leading to social and psychological empowerment of the entrepreneurs, and (3) skills, knowledge, and experience that are also strengthening their human capital in the job market. Economic sustainability of the businesses established is not the primary goal in these undertakings. The article suggests that due to the three factors before mentioned that the notion of social innovation in senior entrepreneurship might best be understood as improving the well-being and quality of life of the entrepreneurs themselves. Senior entrepreneurship can be an adequate response to the challenges of the aging population. However, due to the low rates of unemployment, the idea of becoming a senior entrepreneur appears a little tempting.

## Introduction

Most of the European countries are struggling to find ways to tackle the demographic consequences of the aging workforce. Political strategies (e.g., EU Stockholm-target, the Europe 2020 strategy, the European Year for Active Aging and Solidarity between Generations) promote longer working careers and therefore the increase of participation of older individuals in gainful employment. In this context, senior entrepreneurship seems to represent un unrevealed potential for economic prosperity for older age. Affected by the demographic changes are also the entrepreneurial activities in Europe, in which one notices a current rise of older entrepreneurs (Kautonen, 2013). The Organization for Economic Co-operation and Development (OECD), together with the European Commission, seeing a possibility of accommodating older workers to new roles, published a Policy Brief “Senior Entrepreneurship” to encourage policy-makers to promote entrepreneurship of older individuals by creating favorable environments and frameworks [OECD (Organisation for Economic Co-operation Development) and European Commission, 2012].

Social innovation, on the other hand, is seen by the EU as a “driver for change” (European Commission, 2014). It is presented as a solution to social, political, demographic, or economic problems where other solutions are not available or are not effective. Furthermore, social innovation is also perceived as a mechanism for achieving systemic social change. It is seen as a way of tackling the underlying roots of social problems rather than just alleviating the symptoms.

Senior entrepreneurship could be viewed from two perspectives. On the one hand, senior entrepreneurs are innovators as they go against the Schumpeterian ideal type of entrepreneur, i.e., a young, preferably male, often white, dynamic, innovative, risk-taking, opportunity grabbing individual, who is entirely responsible for his or her success and failure (see: Brockling, 2007; Ainsworth and Hardy, 2008; Franke, 2012). Taking on roles socially prescribed for the younger generations, senior entrepreneurs overcome stagnant models of activities in older age and contribute to the creation of new, innovative solutions to unemployment, underemployment, social exclusion, or poverty. On the other hand, senior entrepreneurs seem to have more socially oriented goals in the performance of their enterprises, which they fulfill in the form of social entrepreneurship (Stumbitz, 2013).

The paper provides a theoretical input on the discourse on the capabilities of social innovation for creating sustainable social change in the area of senior entrepreneurship. Furthermore, the paper delivers empirical examples from an ongoing research project^1^ on senior entrepreneurship in Germany and Poland, where interviews with experts and older entrepreneurs were carried out. The project inspects the transitions and trajectories of adults who decide to become self-employed later in their careers (after 45/50 years of age). The life course perspective allows to track the critical paths and moments, both in professional, as well as in private life trajectories, which determine the decision to become an entrepreneur in later life. Moreover, the study looks at future perspectives of senior entrepreneurs and their plans and projections for the working life after the retirement age and attempts to determine the factors responsible for the successful prolongation of self-employment after the pension age.

This paper aims to evaluate the potential of senior entrepreneurship to become a sustainable social innovation solution for aging population on the example of business incubators for senior entrepreneurship in Poland^2^. It attempts to answer the following questions:

- In what way does a senior entrepreneurship incubator constitute a social innovation for an aging population? What are the innovative characteristics of such an incubator?
- What are the opportunities and limitations of business incubators for senior entrepreneurship?
- What is the potential of senior entrepreneurship incubators to contribute to a sustainable transformation in the socio-economic situation (improvement in subjective well-being, health, social support, financial situation, social connectedness) of older adults?

These questions are answered based on the analysis of three case studies of incubators for senior entrepreneurship in three polish cities: Warszawa, Gdynia, and Gdansk. The empirical material chosen for this analysis consists of in-depth interviews with experts (*N* = 6) and senior entrepreneurs (*N* = 4), who participated in the projects of incubators. Secondly, the analysis of final reports and evaluations from the implementation of the three incubators serves as a secondary data source.

The paper consists of four sections. Firstly, a theoretical background to the theme of social innovation and social entrepreneurship is introduced, where most important concepts are characterized, as well as some examples of ways to evaluate social innovation for an aging population. Secondly, the case studies of senior entrepreneurship incubators are shown, where details about this study's methodology, implementation and innovative character of these projects is presented. The third section is the discussion, where the economic and social impacts and sustainability of these models are discussed with reference to the main research questions. A brief section with conclusions closes the paper.

## Theory and Practice of Social Innovation for an Aging Population

### The Theory of Social Innovation

“Social innovation” as a keyword has increasingly been used in political debates (e.g., Europe 2020 strategy) to promote new solutions for social challenges. However, there is a broad range of what constitutes social innovation, and concomitantly there is a lack of “a universally accepted definition of social innovation and ambiguity surrounds the term” (de Bruin, 2012, p. 373).

Innovation is inherently linked to Schumpeter's (1934) notion of “combination of production factors” and of “creative destruction” (Schumpeter, 1950 [1942]), considering the continuous mechanism of replacing established practices an inherent feature of capitalism. Despite Schumpeter's inclusion of market innovations as well as legal and institutional innovations (Rammert, 2010), the common notion of “creative destruction” has remained omnipresent with a strong focus on technology (Zapf, 1989), which is reflected in the general definition of the [OECD (Organisation for Economic Co-operation Development) and Eurostat, 2018] (“Oslo Manual”) of innovation. However, the shift from post-industrial societies toward knowledge and service-based economies brought social innovation into the limelight of academic as well as public discourse (Schwarz et al., 2008). Technological innovation alone had increasingly been considered insufficient to offer solutions to changes in societies and economies such as aging populations or cuts in the social budgets with ongoing long-term unemployment (Howaldt and Jacobsen, 2010). Social innovation seemed to be the response to these unmet needs. This paper adopts a definition by Howaldt and Jacobsen (2010), who define social innovation as a new combination of social practices in social contexts by a constellation of stakeholders pursuing the goal of tackling problems in an improved manner compared with established social practices. In addition, social innovations need to be socially acknowledged and broadly diffused in society or certain social areas. Further, they are transformed in the context and institutionalized as new social practice. This definition comprises social innovations whether they are marketable or non-profit (ibid., 88–89). Moreover, social innovations transcend the concept of inventions as they need to be turned into “practical approaches” (Evers et al., 2014, p. 11). While for analytical purposes, there is a need to develop distinctive stages or life-cycles (Murray et al., 2010; Bates, 2012) in the social innovation process, in reality, these are rather messy and unorderly. Moreover, not all stages of such life-cycles of social innovations are defining characteristics as (Howaldt and Schwarz, 2016, p. 63) argue.

Inherent to the concept of social innovation is the centrality of values as social innovations—according to the definition given above—is oriented to overcoming problems left unsolved before (Mulgan et al., 2007; Naegele and Heinze, 2012). While some scholars prefer a definition of the concept without including the outcome of social innovation as necessarily good (Degelsegger and Kesselring, 2012; Pue et al., 2016), others, on the other hand, even go so far to include the criterion of “enhancing society's capacity to act” (Hubert et al., 2010) or to bring about change to social relations (Martinelli, 2012). However, they all share the discard of the Schumpeterian ideal of (private) market competition since the goal of social innovation is essentially considered a contribution to the common good (including, e.g., social capital) in contrast to technological innovation (e.g., gerontechnologies and smart technologies including automation and service robotics) (Klimczuk, 2015b).

The mapping and measuring of social innovation practices to analyze the potential success and sustainability or the outcome of practices of social innovation has been diverse, analogous to the various perspectives and social fields the innovations occur. Indicators and tools for the profit sector appear to be rather problematic for the social sector as financial performance is not at the center of social sector activity (Weaver and Kemp, 2017). Moreover, as several processes have not been identified, the given measurable factors might stress the already known over the immeasurable, yet relevant (Weaver and Kemp, 2017). Nonetheless, it has not been identified how social inventions become social innovations or how social practices become sustainable (Howaldt and Schwarz, 2016).

Just as innovation is key in the classical understanding of entrepreneurship according to Schumpeter, social innovation is central to entrepreneurship and social entrepreneurship. Their link gathered momentum when Muhammad Yunus was awarded the Nobel Peace Prize in 2006 with his microfinancing program highlighting the role of pioneers such as “social entrepreneurs.” The following section outlines the relationship between social entrepreneurship as an example and drivers for social innovation at the same time.

### Entrepreneurship and Social Innovation

With the notion of contribution to the common good as one of the core criteria of social innovation, the connection to social entrepreneurship becomes quite apparent. Sharing this goal, social entrepreneurs aim to create social value as opposed to personal or shareholder wealth (Noruzi et al., 2010). Furthermore, social entrepreneurs make use of social innovation as they create new combinations of services, products, or organizations (Defourny and Nyssens, 2010). Thereby, social entrepreneurship is rather a collective endeavor: created by collective actors in the same social system through interactive learning, not individually by each social entrepreneur (McElroy, 2002; Dawson and Daniel, 2010).

The term *social entrepreneur* was used in 1972 by Joseph Banks' *The Sociology of Social Movements* precisely referring to the same definition of social innovation as to address unmet social needs and additionally addressing business challenges (El Ebrashi, 2013). Social entrepreneurs do need to perform financially, yet the social motive is the real driving force behind their business endeavor (Austin, 2006). Social enterprises often work between the private and public sectors, meeting the welfare needs of citizens affected by social and economic inequalities (Shaw and de Bruin, 2013).

Thus, social entrepreneurship can be defined as “the activities and processes undertaken to discover, define, and exploit opportunities in order to enhance social wealth by creating new ventures or managing existing organizations in an innovative manner” (Zahra et al., 2009, p. 519). Even more so, they need to bring about a lasting change (Nicholls, 2006): social entrepreneurship needs to fulfill the criterion of sustainability (Austin, 2006; Robinson, 2006; Martin and Osberg, 2007). Social entrepreneurship–similarly to social innovation–can become the strongest where established organizations and institutions are weak or absent (Desa, 2012). This is particularly the case if the change is more drastic which the social enterprise brings about (Moore et al., 2012).

According to the criteria for social innovation described above, social enterprises tend to rely on finding “solutions” to social challenges. Recently, the shift in demographic structures is among the most remarkable dynamics in every society leading to several unmet needs in so-called “aging societies.” Social entrepreneurship seeks to find a response to various age-related challenges. In addition, senior entrepreneurship could provide an innovative idea to include older individuals as senior innovators and part of the emerging stream in creating social innovations. The following section gives an overview of social innovations for the aging population and highlights two initiatives in Europe in the field and their attempt in measuring and evaluating successful social innovations.

### Social Innovation for an Aging Population

At present, population aging in Europe has now become a significant concern in political debates on future economies, especially concerning national debt. Concomitantly, this trend has impacted the discourse on “social innovation.” With fewer young people entering the labor market, negative outlooks predict new competitive situations on the labor market and a possible “innovation lack” in some industries in the EU. Population aging can, therefore, be considered an obstacle to developing new types of social innovations. However, aging populations can also be perceived as a dynamic driver for social innovations addressing the increasing needs of new products or services (Naegele and Heinze, 2012; Khan, 2013; Foster and Walker, 2015). Apart from revising social norms of aging, enterprises, for example, will have to incorporate specific forms of dealing with older customers and communication abilities spanning the generations (Khan, 2013). With demographic change manifesting itself in general aging of society the question is now, what are promising patterns of social innovations concerning the needs of older individuals? Moreover, what can be the role of senior entrepreneurs?

Senior entrepreneurship is only very slowly finding its place in more general theory on age-related social innovations. Nonetheless, the phenomenon of mature entrepreneurship has been studied empirically for over three decades and explored, for example, the perception and differences between younger and older entrepreneurs (Kautonen, 2008). Among the obstacles faced by older entrepreneurs, few financial and social resources, problems receiving preferable credits, ageism and age discrimination, lack of information and communication technology (ICT) skills or health problems may create barriers to entry into successful self-employment (Zissimopoulos and Karoly, 2007; Franke, 2012; Kautonen, 2013; RKW, 2013). However, some specific advantages of senior entrepreneurship have been revealed as work and industry experience, more developed social networks, higher technical and managerial skills, as well as a stronger financial position compared to younger persons (ibid.). In addition, senior entrepreneurship activities often take part in the field of social innovations, especially catering to senior needs, and may benefit from the seriousness and “speaking the same language” as older clients (Franke, 2012). However, the combination of social innovation, social entrepreneurship, and senior innovators seems scarcely noticed.

In the last decade, the EU has supported research on social innovation and active aging. For example, the project FUTURAGE in the EU's Seventh Framework Programme (09/2009-12/2011) aimed to create a Road Map to guide European research on aging and health for the next 15–20 years. During these 2 years, “the FUTURAGE project focused on the necessity for a new vision of aging and innovative ways to develop the science of aging” (Futurage, 2011a, p. 2). From a multi-disciplinary perspective, the consortium identified seven key dimensions (Futurage, 2011b, p. 9) concerning active aging over the life course such as healthy aging, home and community, Biogerontology, unequal aging, social protection, inclusion and social participation, and mental capacity.

Even with an implicit perspective on “social innovation,” the FUTURAGE underlined the importance of the participation of older individuals and the inclusion of aging in the strategic innovation agenda of the European Institute of Innovation and Technology (EIT) (Futurage, 2011a). In addition, the Road Map significantly influenced the “2012 European Year for Active Aging and Solidarity Between Generations.” However, the potential of older adults in terms of senior entrepreneurship has been neglected.

In 2011, the European Innovation Partnership on Active and Healthy Aging (EIP on AHA) had been established within the EU 2020-strategy as an initiative launched by the European Commission. The initiative of EIP on AHA started with the idea to enable products and stimulate services in the business market for digital solutions in the health care sector (European Commission, 2018).

In its FAQ-paper, the EIP on AHA focused on digital innovations and digital market and made no coherent link to the theoretical concept of “social innovation.” Referring to a broad definition of social innovation complementary to technological innovation, solutions are promoted at the individual, social, and social policy level creating market opportunities for businesses driven by a societal challenge, for example, growing relevance of non-communicable diseases (Richardson et al., 2014). The EIP on AHA, therefore, refers to the Schumpeterian ideal of (private) market competition with a focus on technological innovation rather than new social practices or the inclusion of senior innovators.

A landmark model linked to scientific knowledge and research on age-related social innovation was the InnovAge Project (12/2012-11/2015). The project was comprising a three-year-program to “developing and testing, as well as surveying and cataloging, social innovations that will have a solid impact on improving the quality of life and well-being of older people” (InnovAge, 2016). The project concentrated on the active participation and co-production of older individuals in the developing process. Apart from four tried and tested social innovations, the project further aimed to provide a definition and assessment criteria for age-related social innovations:

“Social innovations are ideas, products, services or models that are new, or being applied in new contexts, and which are designed to improve the well-being and quality of life of people as they age.”

This broad definition underlines, in particular, the purpose of social innovations but disregards the question of the new combination of social practices. For the assessment, the project developed a balanced scorecard, which consists of four criteria (Walker, 2014). To be considered a social innovation, all four criteria—social and economic impact, sustainability, tolerance, and implementation—have to be met. The criteria “social and economic impact” refers to health economic indicators such as systems costs savings due to prevention. Sustainability in the second field means the potential of a continuing product or service with regard to the local infrastructure, costs, and demand. Acceptability of stakeholder and user-friendliness (e.g., co-design) are also key dimensions of social innovations especially with regard to the feasibility of technology-based solutions. The fourth criteria “implementation” deals with the sensitive question of the success of social innovations when it comes to the users, related skills, and the ability to transfer this innovation into different contexts (e.g., by pilot projects).

In addition to the balanced scorecard, the project InnovAge developed eight distinct domains to represent various categories of the aims of social innovation on active and healthy aging:

1. Promoting physical activity among older people.
2. Improving access and provision of health or social care (incl. support for carers).
3. Prevention and management of long-term health conditions (e.g., non-communicable diseases such as diabetes and dementia).
4. Reducing social isolation and preventing loneliness.
5. Providing social support and building social cohesion (e.g., participating in a social or leisure event or performing a daily living task for someone in need).
6. Using ICT to connect (building social connections and improved access to health and social care).
7. Promoting lifelong learning, skills, and paid employment.
8. Intergenerational activity is promoting solidarity.

These dimensions are related to the strategy for healthy aging from the World Health Organization [WHO (World Health Organization), 2012] and reflect an integrated picture of age-related social innovations by providing an analytical tool for complex interventions, services, and products in aging societies. The focus is on health-related changes with age but also on social cohesion, labor, learning, and intergenerational solidarity. ICT is mentioned as one key dimension but might also be a catalyst for other dimensions.

For measuring outcomes and impact of social innovation, InnovAge suggests the idea of “process evaluation” and immediate health and well-being measures such as activities and instrumental activities of daily living (ADL/IADL), disability measure, self-rated health or, for example, proximal measure as weight loss, level of physical activity, or vulnerability. The consortium then developed specific domains for healthy life years with regard to the improvement of health/functions, quality of life and participation/social cohesion (Walker, 2014).

With regard to the participation-strategy of the project, InnovAge also published “Guidelines on involving older people in social innovation development.” The guidelines emphasize the essential active co-production of older individuals in the “planning, development, and implementation of social innovations” (InnovAge, 2016). Methods, which are presented in these guidelines are, for example, surveys and questionnaires, focus groups, workshops, or advisory boards. However, besides questions of success, the project also revealed possible obstacles for social innovations. These barriers can be related to stakeholders and users (especially minors, or those with reduced mental capacity), lack of (digital) literacy, security and trust issues, the risks of highly localized initiatives reaching the economies of scale to generate a sustainable business plan, and; developing an evidence base of efficacy (ibid.).

Another approach of assessing age-related social innovation, derived from the Age Platform Europe, an EU network of non-profit organizations of and for older people established in 2001. Age Platform Europe launched in 2013 a project on social innovation, which “aims at helping policy-makers to get an overview of potential policy needs, funders to receive ideas for potential investments, social entrepreneurs to gain inspirations for potential business ideas and social innovation incubators to improve their service…(and to) create a platform of ideas for social innovations which can be scaled-up tackling the challenges of aging” (Age Platform Europe, 2013). This was one of the first stimuli mentioning at least social entrepreneurship as one dimension of social innovation.

Age Platform Europe together with the other partners created a “Social Innovation in Aging: the European Award,” sponsored by the King Baudouin Foundation, aimed to encourage social innovators all across the EU to present their specific initiatives in the field of active and healthy aging (ibid.). In addition, 20 case studies of 220 applicants have been chosen for a deeper analysis to identify specific patterns of age-related innovation patterns.

Based on the application of different social innovation projects, the consortium identified so-called “guiding principles” for social innovation (Kesselring et al., 2014, p. 165–166). These core principles were as followed:

1. Successful innovations need to be simple and clear in their idea to prevent confusion from supporters and clients.
2. Successful innovations show positive user and volunteer experience and benefits.
3. Innovative initiatives recognize societal challenges and the need to innovate social systems instead of simply compensating for their shortcomings.
4. Social innovation should act resource-oriented on user capacities instead of deficits.
5. Social innovation respects active participation and older individuals as co-creators.
6. Social innovation refers to the importance of voluntary work and the new roles of volunteers in terms of benefits for clients, themselves, and the community.
7. Social innovation means a constant process of learning.
8. Evidence-based technologies, practices, and services drive innovative solutions.
9. Increased observability blends service provision with raising public awareness.
10. A combination of social interaction and technology offers the potential for social innovation (ICT, social networks, assistive technologies, etc.).
11. Use of more sophisticated and impact-focused evaluation tools is required (instead of less flexible measures).
12. Extension and diversification of cooperation networks support success.

European Policies seem keen to find new solutions for tackling the aging of society by supporting several initiatives on social innovation in aging. Similar to the general still existing fuzziness of “social innovation,” also the question of social innovation in an aging population arises in terms of definition, specific demands, assessment criteria, and measures for output and impact.

To summarize the activities mentioned above, most notions of social innovation for an aging population entail the idea of catalyzing demographic dynamics, participation, and the improvement of living conditions of older adults in different regions. While the concept of social innovation itself is multifaceted, its interpretation regarding an aging population also comprises a wide range of new types of organizations (e.g., age management in companies), services (e.g., integral forms of co-operation in care) as well as new patterns of social practices (e.g., voluntary work, multigenerational housing). The described projects illustrate a strong focus in the current social, political, and economic discourse on active aging, health, care, intergenerational relations, and active participation also concerning the community level. However, there is still a lack of flexible measures dealing with the complex framework and structure of social innovation activities. Most of the tools are developed for economic and accounting context and rather inappropriate to analyze social impacts. Another consideration is that social innovation for aging depends on the exploration and establishment of cooperation. In this context, platforms for interaction and networks are necessary to enable supportive collaborations. It can also be concluded that social innovation from the employers' perspective regarding age management, social policy innovation or senior entrepreneurship seem neglected topics. Senior entrepreneurship does not stand out in the sense of a technology-based innovation concept, but it holds potential for social innovations, for example, in the field of social entrepreneurship.

## Senior Entrepreneurship Incubators as Models of Social Innovation for an Aging Population

### Senior Entrepreneurship Incubators in Poland

Entrepreneurship or business incubators are “organizations designed to accelerate the growth and success of entrepreneurial companies through an array of business support resources and services that could include physical space, capital, coaching, common services, and networking connections” (Small Business Encyclopedia, 2018). As such, these organizations (either public or for profit) are a well-known phenomenon in many countries and especially vivid in the branch of new technologies, where mostly technological start-ups are being sponsored. Thus, most of such initiatives are being targeted at younger persons—the ideal type of an entrepreneur (Matricano, 2018). However, some efforts, mostly in Europe, have started to encourage and support senior entrepreneurship. The EU was one of the first organizations to create policy initiatives aimed at stimulating entrepreneurship among older people (Stypinska, 2018). “SeniorEnterprise.ie” in Ireland is an EU-supported initiative through INTERREG IVB NWE, specifically designed to encourage a greater involvement with enterprise by those aged over 50. In this way, senior entrepreneurship addresses the concerns of the many European countries with regard to the challenges posed by an aging population and the need to increase productivity, competitiveness, and entrepreneurial activity across the EU (Isele and Rogoff, 2014). As suggested by Klimczuk, (2015a, p. 4): “Social innovations in Poland are considered mainly in the context of the social economy and social entrepreneurship. Thus, their development and implementation primarily relate to social work and solving social problems such as unemployment, poverty, integration, and employment of people with disabilities, the reduction of the social exclusion, homelessness, and the fight against addiction.” Incubators for senior enterprises, as organizations or projects addressing the unemployment, risk of poverty and social isolation of older persons, are innovative ways for addressing these challenges and have the potential for becoming social innovations worth spreading.

Within the framework of EU funds many projects aiming at increasing economic activity in the age group 45/50–65 were carried out (Kubicki, 2012), however, the specificity of targeting this very group with the offer of starting a business was very rare. Most of the projects targeted at unemployed persons age 45/50 were aiming at reintroducing them to the labor market as employees (through training etc.), whereas projects promoting starting a company were not age-specific (or explicitly targeted at young persons) and thus quite often omitted the older age groups due to stereotypical preconceptions that entrepreneurship is for the young only. Thus, a specific niche appeared as space where projects bringing the two dimensions could successfully apply for EU funds.

The starting point for the implementation of these project—incubators in years 2010–2014 were diagnosed problem areas, such as: low professional activity of people aged 50+ in Poland, rapidly progressing population aging process, lack of opportunities to return to the labor market after losing employment, lack of tools for vocational counselors dedicated to work with people 50+ and lack of an appropriate offer of support in the field of entrepreneurial incubation (Kubicki, 2012). The phenomenon of a decline in economic activity was additionally intensified by the pension reform introduced in Poland in 2012, which extended the retirement age to 67 for men and women^3^. Hence the intense interest in addressing this age group. However, the exact number of similar projects carried out in Poland is not available, and thus the presented cases constitute a choice based on the above criteria and the suggestions from experts.

### Case Studies Selection

The following section describes the empirical case studies of three incubators for senior entrepreneurship which were operating in Poland between 2010 and 2014 in three cities: Warszawa, Gdansk, and Gdynia. All three were subsidized with public money with a major contribution from the EU funds for social projects. The case studies presented here were chosen according to the following criteria:

- The primary goal of the project was to create an incubator-type of support for the group of persons 45/50 plus with the intention to become self-employed.
- The projects are finished.
- Visibility and accessibility of information about the incubators on the Internet and through expert knowledge (expert interviews).

The selection of the case studies according to the above criteria allowed to study those projects which could be compared due to the similar structure of funding, management, target groups, and aims. Moreover, they delivered rich data (evaluation reports, experts' evaluations, interviews with participants and experts) which allows a deep and thorough analysis. To the best knowledge of the authors, the case studies presented in the paper represent the whole sample, and no further cases could be found, which fulfilled the above criteria. Thus, it was concluded that the selection strategy allowed to achieve a sample and data saturation, where all possible case studies fulfilling the criteria are taken into consideration and on the basis of the data that have been collected and analyzed hitherto, further data collection is unnecessary.

The analysis presented in this article does not constitute a systematic evaluation of the projects in the strict sense of evaluation of social innovation with the use of a rigid set of indicators (Riess, 2010), as those have been done by appropriate institutions after the duration of the project. The analysis presented here follows flexibly recommendations from the InnovAge project (InnovAge, 2016) to evaluate the social innovation projects but concentrates only on chosen aspects as those which correspond with the research questions of the paper. Thus, the three case studies of incubators for entrepreneurs 45/50+ from Poland are described with regard to four dimensions (a) goals of the project and implementation (understood as actions, measures, effort, etc.); (b) outcomes (understood as immediate results of the project), (c) innovative dimensions (e.g., participation, user-friendliness). The next section, a synthesis of all the models, is presented in relation to (d) social and economic impacts of the projects (changes in society and in the institution, changes in behavior, attitude, relationships among the target group and in the institution) (Riess, 2010) and their sustainability (potential for contributing to a long-term social change). The empirical material used for describing and analyzing the case studies of incubators for senior entrepreneurship includes: self-reporting materials (descriptions of the models developed), external evaluation of projects, expert interviews (*N* = 6), and interviews with participants—senior entrepreneurs (*N* = 4)^4^. The experts were either external actors, not directly involved in the projects, or leaders of institutions implementing the projects. The experts were selected according to their knowledge, experience, and proximity to the projects of incubators, i.e., the level of engagement in the carrying out of projects. The experts represent the following functions: expert 3: external evaluator for the case study II, academic expert in gerontology at the Pedagogical University in Krakow, expert 6: external evaluator for the case study III and academic professional at the Warsaw School of Economics in the area of gerontology; expert 10: project leader for the case study I, developed the idea for the incubator and managed its implementation, based in Warsaw; expert 11: project leader and developer for case study II, based in Gdansk, expert 12: the president of Economic Foundation (Fundacja Gospodarcza, 2014) in Gdynia, responsible for formal implementation in the case study III; expert 13: employee in Economic Foundation, responsible for implementation and running of the incubator in case study III. Three experts expressed their willingness to remain anonymous, and hence we adopted this approach to the rest of the interviewee partners as well. The entrepreneurs interviewed for this study were all participants of the incubators, who participated fully in at least one model of the incubator (one participant took part in two projects). The entrepreneurs 13, 17, and 19 were female; the entrepreneur 15 was male. The interviews were carried out within the framework of the research project MOMENT, which brief description follows.

### The Research Project MOMENT

The research project MOMENT is an ongoing project (duration: 01/2017-12/2019) funded by the German Research Association (DFG). It examines the process of becoming an entrepreneur in later life in relation to previous career paths in order to establish how life course experiences determine the shift to self-employment. It explores a basic question about how individual and institutional conditioning impacts on the process of making mature entrepreneurs. The thesis proposed is that the entrepreneurial motivations and activities of older adults are the outcome of a dynamic and reciprocal relationship between their personal and occupational life paths n the one hand, and societal and structural feedback received from institutional (formal and informal) actors on the other hand.

The project methodology is based on individual in-depth interviews with entrepreneurs 45 years and older, as well as expert stakeholders in Poland and Germany. In Germany, interviews have been conducted in the West and the East of the country to obtain the most diversity regarding economic power and industry. In Poland, two voivodeships (Małopolska and Pomerania with their urban capitals of Gdansk and Kraków) and one single urban area (Warsaw) were chosen as fieldwork regions. Małopolska region can be characterized by relatively high labor market activity of older persons in comparison to the average in Poland and has strongly embedded support structures for the activation of older persons in the labor market. Pomeranian Voivodeship was chosen as it has already implemented successful projects of supporting older persons in founding their business activity. Thirdly, Warsaw as the largest urban area in Poland was chosen due to the high propensity of entrepreneurial activity in the area, easily accessible structures of support and assistance for entrepreneurs, as well as lower levels of unemployment among older persons.

The empirical material gathered until now comprises: qualitative interviews with 13 expert stakeholders^5^ (Meuser and Nagel, 1994) and 19 interviews with senior entrepreneurs in Poland. The interviews have been transcribed and analyzed with the method of qualitative content analysis according to Mayring (2000). The empirical data has been analyzed according to the common scientific praxis in sociological research (anonymous transcriptions, rigorous data analysis with the assistance of software MAXQDA).

## Case Studies

### The Case I “Incubator of Mature Entrepreneurship” (Warsaw, Mazovia Voivodship)

#### The Project: Goals and Implementation

The project “Incubator of Mature Entrepreneurship” co-financed from EU funds under the European Social Fund was implemented by the “Cooperation Fund” Foundation in partnership with the Foundation for the Promotion of Social Initiatives POLPROM^9^. The project aimed to increase the entrepreneurship of people aged 45+ in the province Mazovia (Mazowsze) through training and advisory support of 50 persons, and through financial assistance for 35 persons intending to start a business. Project activities were carried out from December 2010 until December 2012, and they were guided by the slogan “It is never too late for success” (Fundacja POLPROM, 2012). The starting point for the Incubator was to develop a “Competence Profile of an Entrepreneur,” which would be a guiding principle for recruitment phase, as well as the training and counseling phase for the participants. The profile included 17 competencies in four categories: business competence, social competence, personal competence (soft competences), as well as hard competence category of professional skills (hard competencies). The project's recruitment process consisted of two stages—analysis of applications and individual interviews. In the first stage, the original business idea was evaluated, including market analysis, characteristics of potential clients, and competition, as well as an estimate of the total investment and resources; experience, knowledge, and predispositions related to a business idea, as well as motivation, to participate in the project. Finally, 50 persons were accepted, including 27 women and 23 men. Most participants were professionally inactive (32 people), but there were also 13 people employed and at risk of losing their job and five unemployed people. Six participants with disabilities were accepted.

The implementation phase of the project included several types of support: training and counseling, providing office space, where participants could meet and carry out their different activities, and financial support. The training phase involved: psychoeducational workshops (16 h), basic business training (64 h), specialized optional training (20 h), business consulting (12 h), career counseling (4 h), educational consulting (6 h), interventional business consulting (18 h, available in the first year of running a business). The financial support included: one-time subsidy for setting up a business (up to PLN 40,000,~10,000 euro)−36 people; “bridge support” (PLN 1,000/month)−36 people; extended bridging support (PLN 1,000/month)−12 people.

#### Outcomes^6^

The tailored model of training/counseling approach used in the project proved to be effective as shown by the level of satisfaction of participants, as well as the implementation of the pre-defined indicators. All participants finished six-month training and consultancy and prepared business plans. However, the scope of training was also problematic due to family obligations or time restrictions. Moreover, the fact that the participants needed to remain in training for more than half a year was a limitation: “This is both a lot and little, I think that it is not enough, and for people and for me too, who want to set up the business the time is stretched, half a year is very troublesome because imagine: people are without work, usually, on the support of spouses, they often have children” (ENTR 13). In the end, 48 business plans (96%) were rated positively. Due to budgetary restrictions, only 36 persons received subsidies for starting a business.

#### Innovation and Participation

The innovative dimension of the project could be observed in several specific features of this project in comparison to other projects supporting persons wanting to start the business. It could be briefly summarized in the definition of the Incubator for Mature Entrepreneurs itself: “The Incubator is a method of supporting people aged 45+, which creates the best conditions for acquiring knowledge, acquiring competences and taking the first steps toward the enterprise parts—minimizes stress, mobilizes, increases the effectiveness of actions training and consulting and facilitates running the company in the first years' activities. The incubator responds fully to all diagnosed educational needs of this target group” (Fundacja POLPROM, 2012). Another innovative dimension of this project was that the project and office spaces etc. was adapted to the needs of persons with disabilities, which was intended to provide more inclusion for the participants of a certain age. This model constitutes a social innovation in three areas indicated by the guidelines of project InnovAge: reducing social isolation and preventing loneliness, providing social support, and building social cohesion, and promoting lifelong learning, skills, and paid employment (InnovAge, 2016).

### The Case II: Pomorska Fabryka Designu “Pomeranian Design Factory” (Gdansk, Pomeranian Voivodeship)

#### Goals and Implementation

In May 2012, the Gdansk Entrepreneurship Foundation started the implementation of an innovative project “Pomeranian Design Factory.” The project was implemented in partnership with the Poviat Office in Gdansk. The project aimed to extend the age of professional activity of people 50+ residing in the province Pomeranian through actions combining creativity and handcraft skills with entrepreneurship, which was an unusual idea based on a similar project in Switzerland (EXP 11). As part of the model, comprehensive support was provided for people who were unemployed, enabling them to create their own workplace, from which, through performing their hobbies and using their manual skills they could earn a living. The initial number of persons who applied for the program was more than 100, which boiled down to 15 participants after the first evaluation and after the final Assessment Center Methodology of recruitment. Moreover, a product distribution tool was launched (an online sales platform), where the participants were allowed to operate under a shared brand (pol. “Sto palców”; eng. “Hundred fingers”). The incubator model consisted of 9 modules: (1) Recruitment process of the participants and supporting team, (2) Training module for the participants and the support team, (3) Advisory module for the participants—design, business, and accounting-legal advice, (4) The business model of the undertaking, (5) Guide to building an online sales platform, (6) The marketing strategy of the undertaking, (7) Guide to creating a brand, (8) Organizational and legal model of the brand and online sales platform management, (9) Financial subsidy for starting a business and bridging support (Rzeczkowska-Owczarek et al., 2014).

#### Outcomes

The outcomes of the model developed within the project Pomeranian Design Factory were measured in an external evaluation using the testing result indicators. The monitoring covered 11 stages, and 33 indicators were used. Out of this 11 were achieved at 100%, 14 at more than 100%, and 8 at less than 100%. Most testing results have been achieved without major difficulties. The lowest achievement was observed in the indicator “the sale of products via the online sales platform.” The index refers to this result was implemented only in 28%. (Rzeczkowska-Owczarek et al., 2014, p. 17). According to one of the interview partners, this problem did not result from the low interest in the brand products, as the sales ratio at other sale points was reached in 450%, but the reasons for low achievements were rather the result of lack of long-term technical support for its operating (EXP 11). One participant commented it this way: “Because this store was very nice, everything was nice, nice. We learned to handle it, but it was not so that someone would run this shop, it would not be, because it would be very labor-intensive, this person would she could not drive her business anymore, right?” (ENTR 19).

Each participant of the project received financial and non-financial support in the form of business and design-creative training, as well as design, business and accounting-legal consulting (Rzeczkowska-Owczarek et al., 2014). The classes with designers, who played the role of both advisers and mentors, were perceived as especially valuable. Three out of five participants pointed out that the most important (“turning point”) element of their participation in the project was the cooperation with the designers (ibid.).

In the effect of the project, all participants received the financial subsidy (~16,000 PLN = 4,000 Euro) for business and opened their own companies. The financial support was recognized as the most crucial element of the whole project and as many as 10 of the 14 project participants (71%) indicated that they would not decide to open own business if they did not receive financial support. The financial subsidy was received positively by the participants, but some critique was also expressed “we got some money, now I will not tell you exactly how much but a dozen or so thousand and it was a bit bad because they told us to spend the money immediately, and we didn't yet know what we needed…Moreover, it terribly annoyed me; I do not like spending money aimlessly (…) if I got money in cash, then I could spend it successively when there would be a need and not so…” (ENTR 17). For another person, the money was actually the motive she did open the business: “it was a good start, that extra money, I could buy the materials and things I needed” (ENTR 19).

#### Innovation and Participation

The project had several elements which were of an innovative character. First of all, it was the first project addressed to a very particular group of persons 50+, namely those who were highly gifted in their craftsmen skills. Secondly, careful recruitment of the participants was carried out with the use of a method—Assessment Center^7^, which had never been used so far to recruit unemployed persons to activation programs. In contrast to the traditional methods used, this tool allowed to verify the competences and identify the strengths of the participants, as well as areas where further assistance and development is needed. The external evaluation showed that it was more effective by selecting participants who were able to meet the challenges posed by the model (Rzeczkowska-Owczarek et al., 2014). Also, according to an expert in the field of andragogy, “the selection of beneficiaries for the project is crucial. Among the unemployed are those who do not want to take up a job, are unfit to manage their own venture, are finally claimable and helpless—this project should not be addressed to people over 50 with such traits” (EXP 3). Another innovative element of the model was a dedicated online sales platform and operating under a shared brand. Relieving the participants from the obligation to create and manage their own online store allowed to limit the bureaucratic activities and allowed them to focus on the production of designer products. The platform was supposed to stay active after the end of the project and be accessible to those who wish to sell their products there. However, the sustainability of this solution needs to be questioned, since after 4 years since the end of the project the platform is not any more actively used by the project participants (EXP 11).

### The Case III “Mature Entrepreneurship: An Innovative Model of Entrepreneurial Pre-incubation of People 50+” (Gdynia, Pomeranian Voivodeship)

#### Goals and Implementation

Project “Mature entrepreneurship: an innovative model of entrepreneurial pre-incubation of people 50+” was carried out from February 2012 to September 2014 by the Economic Foundation in Gdynia in cooperation with the Poviat Labor Office in Gdynia^8^. The main objective of the project was to develop and test an innovative model of entrepreneurial pre-incubation of people aged 50+ who were economically active, aimed at activating to set up their own business. Sixty-three unemployed people aged 50+ and 4 vocational counselors participated in the project. “Pre-incubation” of entrepreneurship are actions aimed at building the awareness of the older person to such an extent that he/she can decide to start own business. Pre-incubation of entrepreneurship does not assume that everyone has predispositions, competences, and other resources, as well as operate in an environment conducive to running their own business. The aim of pre-incubation is a reliable analysis of the risk and opportunities of running your own company, the results of which may lead to the conclusion that a solution is much more beneficial for a given person will be to remain an employee (Jurek, 2012).

The implementation of an entrepreneurial pre-incubation model of 50+ people consisted of three components: model of consulting, model of conducting training and a multimedia application “Life Design 50+.” Important to underline is that the participants did not receive a financial subsidy to start their companies, what was the practice in two earlier case studies. The counseling model was used to diagnose and supplement the competences of people 50+, which are necessary to be an effective entrepreneur. The model included independent work with the project participants, and they were the basis for designing individual support for each of them. The training model included materials for the implementation of soft skills training in the field of personal development and business training with entrepreneurship and sales skills. This tool was aimed to supplement knowledge of selected areas and support the person in the field of generating business ideas and preparing to run their own business. The Life Design 50+ application (available online at http://www.dojrzalaprzedsiebiorczosc.pl/life-design-50/opis.html) was created as an online tool to structure the process of professional counseling and includes a number of tools and tips useful in the process of estimating the potential of a 50+ person and developing recommendations for effective professional activation. The application consists of three stages closely related to consulting work with a 50+ customer, described in the model of consulting. All intermediate products were based on the skills developed within the framework of the Pyramid project and diagnosed in the study of the needs of people aged 50+ and vocational counselors.

#### Outcomes

The effectiveness of the innovative entrepreneurial pre-incubation model was demonstrated by the high−52% efficiency (the average effectiveness of programs targeted at people aged 50+ in the Pomeranian Voivodeship was 38%). For 60 people who completed the project 21 people took up employment, five people took up a business and five people set up a social co-operative (EXP 13). The training was mostly evaluated very positively by the project participants, however some suggested changes: “These training showed important things, although I say that the training would be more necessary after starting the company, because I had all the theory on this training, but the problems come in during the first months and years of running a company, it is then that questions arise” (ENTR 15). Also, the length of the training was identified as problematic.

The experts interviewed were very optimistic about the outcomes of the project: “We had the effects of dissemination of our model—these are declarations from career advisers from the Pomorskie Voivodeship, but also from other regions of Poland about the willingness to use the entrepreneurial pre-incubation model in their daily work” (EXP 12).

#### Innovation and Participation

The project can be identified as being innovative in a few dimensions. Firstly, the target group—economically inactive persons aged 50+—have not been earlier supported in terms of entrepreneurship, but rather in return to employment. In the majority of projects, only those entrepreneurs who had a specific business idea had benefited from the support in the form of financial subsidy and training. People without knowledge in the area of entrepreneurship and concrete business plan could not submit applications for grants. Supporting entrepreneurial skills and becoming self-employed for this age group was perceived as an innovative way to continue their professional activity. Secondly, this model of entrepreneurial pre-incubation helped in diagnosing the entrepreneurial predispositions of a 50+ person, defining competence gaps and completing them, increasing self-esteem, and positively influencing the attitudes of the recipient of support (Zajaczkowska, 2013). Pre-incubation is a process that anticipates and complements the standard incubation path of companies but does not necessarily impose this solution on the participants. The research carried out in the project showed that mature people require dedicated support, taking into account their specific needs and that is why the preincubation model is more convincing than the standard incubator (EXP 12). The innovative character of the model was appreciated and in October 2014, at the Exhibition Fair in Warsaw, received an award for the winner of the second edition of the competition: “Regatta of Development: Leaders of Innovation and Transnational Cooperation, 2007–2013,” in the Leader of the Innovation category.

## Discussion: Social and Economic Impact and Sustainability of Incubators for Senior Entrepreneurship

This section provides an overall synthesis of the impacts and sustainability of the presented incubators. The projects' impact understood as the long-term effects can be divided into two categories: the financial/economic and social impact. The sustainability can be understood as a long-term social change for the target group and/or society/community. It can be viewed from an individual and/or an institutional perspective.

The financial impact of the incubators was usually relatively limited, as due to the high cost of running a one-person company, most^9^ of the businesses created within the projects needed to close their operation after 1 or 2 years. Almost all the interviewed respondents, both experts, and entrepreneurs assessed that the main culprit of this situation was the structure of social insurance (ZUS; Zakład Ubezpieczen Społecznych, eng. Polish Social Insurance Institution) paid by the self-employed. The reasons for 'losing the complies do not lie in the lack of capabilities of participants to run a firm or their low motivation. Only a few participants of all the incubators were successful financially. For a woman in the Design Factory the project indeed increased her financial stability: “I mean, what has changed, that I have so much now work that I cannot catch up, it has changed (…) at this moment, when someone comes to me, that I, unfortunately, have to say no, because I do not have time anymore” (ENTR 19). In most of the cases, however, the business closures were related to high costs of ZUS (Interview partners 13, 17, 19), which make the financial sustainability of the senior entrepreneurship (which is in most of the cases a one-person-company) very hard to achieve “I closed the company after 2 years, because it's terribly time-consuming and I just cannot afford to pay ZUS, because after 2 years it went into over 1,000 zlotys there, 1,100, basically I would have to pay extra”, “probably all of us knew that after 2 years we would close, but that this ZUS would be impossible” (ENTR 19). Furthermore, all the experts confirmed that the Insurance ZUS is the primary reason for closures “and then this ZUS increases and, unfortunately, it is deadly” (EXP 10). Secondly, the businesses were being closed due to family obligations (caring of older parents) or own health issues. “Well, maybe I gave up too soon because I have a lot of knowledge and willingness, but it was also due to a family situation like that. I have an older mother, 92 years old and if I worked and probably had such a situation, I would probably have to put her in an institution somewhere, right?” (ENTR 13); “Yes, I mean that just because of my and uncle, I do not have such situations that I would have to suspend my business then, right? Because I just cannot, and in this, I am the moment of life that I cannot plan anything simply” (ENTR 19).

The main long-term impact, however, mentioned by the interviewees were not financial, but the personal change and transformation, which proved that the incubators held an empowering role for the older participants in building their own self -esteem: “I know well that people of my age are perceived in a certain way, right? Nobody will believe that I can do it (graphic design) in a modern way. Maybe a little lack of self-confidence? Well, as I started to apply there as well, I did not have any faith that I would get it so well, for me it was incredible, that I got the highest note of women, I did not expect it to be honest” (ENTR 13).

“I could not get over the admiration of how this man made progress just in terms of a more businesslike approach, some more self-confidence, such opening” (EXP 10).

“That I'm independent, when I'm 60, I suddenly became independent, I was never independent before” (ENTR 17).

“In general, this 100 fingers project, it gave me such self-confidence, right? That…I believed in my strength, right? And that someone was standing behind us, that it was such a support, also I have very good memories about this project, and I think…we all think that it gave us a lot, even if we closed these companies? This gave us such a thing…it changed us, very much (…) in my case that I went out more to people, that I made new friends, new contacts, that we continue to grow, because we are learning more—at least some of us, we go to some classes now, also extra” (ENTR 19).

“I felt more appreciated that I have my own company and that's what it was all about…that was definitely important for me” (ENTR 19).

In the project Design Factory, the individual meeting with designers was highly appreciated by the participants, as they managed to increase the self-esteem of the older participants. One of the participants expressed it this way: “They had yy female designers hired, three girls after art schools and they rated the work. And me just…I'm so modest with these sweaters I'm thinking oh God! Some artists will be there, and it turned out that I had the first place…First place, the most points I got from girls, I was so happy! Jesus!” (ENTR 17).

Secondly, participation in the courses, training, and counseling significantly increased their skills and knowledge (human capital). It was confirmed by the external evaluation report of Case II that the participants gained several new soft skills, such as: developing innovative and creative abilities, development of entrepreneurial features, strengthening faith in one's own strength and abilities. The participants admitted it “gave them a lot” (ENTR 17), and that it provided them with knowledge which they used in their future projects and endeavors (not necessarily for economic gains): “but there were girls who could not talk about themselves introduce themselves, they could not…count the business to get something out there, right? And so, the project helped them” (ENTR 17).

The third category in which the participants, as well as experts, saw a long-lasting impact was the building of social capital: new social networks, both in the business, as well as in the private sphere. “Besides, we met each other, we all like each other−12 were girls, 3 boys but they got lost somewhere along the way, and the girls still meet (…), yes, after 5 years we still meet” (ENTR 17). Moreover, the business networks were also strengthened by the media coverage of the project featuring one of the participants: “I was on TV in Gdansk, I was interviewed and from such a newspaper…they asked me that such a newspaper for mature ladies (…) and a reporter, she came to me from Warsaw to interview me and…it got a bit loud because I made a sweater for such a famous lady in Poland and this sweater, and then they started to look who made this sweater. people came to me who was looking for this sweater, but nobody knew who was it because this sweater was in the newspapers and on the Internet portals and everyone…there was such an interest in who did it” (ENTR 19).

The policy suggestion made in the vast literature on senior entrepreneurship underlines that in order to encourage older adults to start their own businesses “the general awareness of third-age entrepreneurship as a viable, positive and attractive late-career option” has to be popularized (Kautonen et al., 2013). In this way, the project Pomeranian Design Factory was extremely successful in gaining public attention and contributing to the improvement of the image of older entrepreneurs in society. The media coverages were positive and pictured the image of older entrepreneurs as very successful. This was furthermore strengthened by professional short videos introducing each entrepreneur and their products.^10^

The sustainability of the senior entrepreneurship could also be observed in the type of some businesses started, especially with regard to the long-term thinking about the needs of aging societies. One of the participants (who previously studies political sciences) opened a podiatry practice seeing a need for this kind of services in the aging population in Poland. It was commented as being almost a revolutionary solution and “it was very brave, because it was like entering a completely new reality (…) because it is not only a cosmetology but already a therapeutic and healing method (…) because diabetes is a civilization illness, so these problems will only increase in the population entering the retirement age (…) so, she thought it would be a very promising and interesting job” (ENTR 13).

The impacts and sustainability of the project also need to be analyzed from the institutional—as opposed to the individual—perspective, i.e., what was the gain/loss for the institutions and organizations implementing the projects. The primary impact identified by all the experts involved (EXP 10, 11, 12, 13) in the projects was an outstanding learning effect from the implementation of the incubators for the project organizers:

“We learned a lot, our employees who worked in this project got really a whole new set of knowledge. Well, if these assessments, creating…thinking about these entrepreneurial traits, creating these scales and so on, meeting with professionals in this area, also meeting with this group 50+, as well as these designers…so far we have never worked with such environment, so entering a new environment, new areas unrelated to business (…) so we have gained a lot of such new knowledge for us and also for the management of a project that was not so simple, which was multilateral—I think it was extremely interesting and a lot of new knowledge. I was very happy” (EXP 11).

Secondly, experts mentioned that the biggest hindrance to the sustainability of these solutions is the short-term thinking of grant givers “only the short-term effects are taken into account (…) To spend money is important and to have a result that after a year nobody resigned, right? And that everyone has kept these companies for at least a year, and nobody is interested in anything anymore. Only what for? Why do these projects at all? If you do not study in the long-term perspective of how these people deal with it. And that if you do not offer this support not only this year, because it seems to me that this support would be needed maybe in a smaller sum, in the longer than just this 1 year” (EXP 10). It was admitted that there is also no systematic monitoring of the activities undertaken by the participants 3 months after the end of the project. Further, the role of indicators used to measure the progress and success of the projects was criticized as not flexible enough (EXP 10). Moreover, the experts remained skeptical about the sustainability of financing this kind of projects in the new financing period of the EU (2013–2020) due to long duration of such incubators and high costs of it, as well as changing priorities in the funding programs, where there is less focus on the labor market measures for older persons.

Another hindrance to the sustainability of the developed model of the incubator in the Case I in Warsaw was that even though model and recommendations exist, they are not used: “it is just a shame that these solutions that are worked out are transferred to the ministry and are lost in some official drawer and then just after a year or two, someone else works out a model in Gdansk” (EXP 10). Sustainability of the model of preincubation in Case I can be seen in the availability of the online tool, which can be used by both persons with the intention to start a business, as well as counselors who work with 50+ clients. The online availability of the platform 4 years after the end of the project is an outstanding result in comparison to other websites, which are no more active and thus the materials (models, instructions, reports, and recommendations) developed within the projects are no more accessible. Therefore, the transfer and the potential for scaling -up of these social innovations was hindered. The expert proposed a solution: “to create some kind of base, a well-made database of national projects such a database of projects in which you could search for such a specific subject, yes, some specific whether it is a model or is there some other type of solution in some field and to make it available. For example, I want to do such a different project, and I have an idea to create an incubator model for mature people, I check if someone has already invented it, right? Because maybe he came up a few years ago and it could be adopted but something to change because something has already changed, right? (EXP 10). The sustainability of the Incubator was thus envisioned, in the creation of a systematic national network of Incubators of Mature Entrepreneurship, which would operate under a single brand and would be recognizable similarly to the Academic Incubators functioning at the universities” (Fundacja Fundusz Współpracy, 2012).

## Conclusions

This paper aimed to illustrate senior entrepreneurship as a potential driver for social innovation in aging societies by presenting three case studies for incubators in Poland. It can be summarized, that the concept of social innovation itself is multifaceted, but its interpretation regarding age-related social innovation seems even more diffuse. So far, most activities related to social innovation for aging populations refer to initiatives regarding health, care, intergenerational relations, and active participation also concerning the community level. However, senior entrepreneurship as a progressive form of integrating seniors in social innovation processes seems a neglected topic. Older entrepreneurs might be especially able to gain older clients and “demographic related” branches in the senior market. The three case studies presented in the paper give evidence for the importance of incubators as a platform for exchange and support for older persons, who decide to become self-employed in later life. However, some limitations and obstacles to the incubators becoming a scalable social innovation solution were also observed.

There is evidence that although the incubators do not sustainably contribute to the improvement of the financial situation of the entrepreneurs 50+, they do hold potential for improvement in: (1) social connectedness (social capital), and thus—decrease in social isolation, loneliness, prevent from social exclusion, (2) personal self-confidence which leads to social and psychological empowerment of the participants, allowing them to participate more fully in social and economic life, “it drags them out of homes,” and (3) skills, knowledge and experience (human capital)—the participation in incubator activities broadened the repertoire of skills and know-how of the participants preparing them to actively pursue further economic activities (not only related to the self-employment status, but also as an employee).

With regard to the economic sustainability of the firms established within the framework of the senior incubators, the results are not as clear-cut. Firstly, some firms still exist, but have low income, usually due to the character of the activity (small input, one-person, based on small capital). Secondly, the majority of firms closed down after 1–2 years of activity, and the reasons for that are mainly related to purely economic/market factors and the characteristic of starting a small business in Poland: the burden of ZUS (social contributions including social insurance) is simply too high. Other factors for closing the firms: the burden of care (usually aging parents), and also own health problems. Therefore, the economic impact of the incubators can be evaluated as relatively short-term and limited to the financial subsidies received from the incubators. However, as shown in the cases of senior entrepreneurs, even after formally closing the enterprise, many of them continued economic activity in another form, which for some of them constituted a steady income.

It can be concluded that the depicted incubators do fulfill the criteria of social innovations with regard to their goals and impacts, that is to: “improve the well-being and quality of life of people as they age” (InnovAge, 2016). It can be assumed that the well-being and quality of life of the participants of incubators were increased due to the identified increases in the three domains: social capital, human capital and the effect of empowerment gained by the participants. According to the so-called “guiding principles” of social innovation, as identified by Kesselring et al. (2014), the incubators for senior enterprisers fulfilled at least 5 out of 12 criteria. Firstly, they were “simple and clear” solutions. The incubators received a very positive public resonance due to a simple idea of supporting older persons in their entrepreneurial activities, which previously had not been done in any systematic way. Secondly, these incubators were based on a thorough analysis of competencies and capabilities of the participants, which was visible in the long and precise recruitment process, especially in the Case II where handcraft skills were a particular focus. Thirdly, active participation of older individuals was seen in the implementation of the project in Case II, where the participants needed to create a common brand for their products. Fourthly, all the incubators reflected the idea that social innovation means a constant process of learning. The training, counseling and further assistance of the senior entrepreneurs provided for a large increase in the human, cultural and social capital of the participants. Lastly, the guidelines define that “A combination of social interaction and technology offers potential for social innovation,” which criteria were met by two incubators (Case II and III), where the technology—Internet platforms (for selling the products, as well as for training and counseling for older adults to entrepreneurship) were one of the core elements of the incubators.

The scale-up possibility for the incubators as a social innovation for the aging population was mentioned by all the experts. However, certain modifications need to be implemented: “I think that this is a very good way to support entrepreneurship such an incubator, only acting according to other principles, right? (…) In other words, greater individualization and the possibility of such more flexible paths in the project for these people” (EXP 10). Moreover, the timing of the implementation of these solutions also plays a significant role in the possibility of spreading social innovation. The problematic situation of the older adults on the labor market in Poland at the time of these projects has in the meantime improved significantly. The employment rates of older population increased and according to many experts, the better economic situation in the labor market and more options for employment decrease the interest and willingness of older adults to start or continue the self-employment, as a steady job position is considered a more attractive alternative to entrepreneurship (EXP 6, EXP 10).

The results from the MOMENT project indicate that even despite the relatively low economic sustainability of the entrepreneurial activities started within the incubator, there is a strong potential for entrepreneurial activities in older age, in the sense of age productivity and social innovation. It needs to be bear in mind, that starting an entrepreneurial activity is a risky endeavor under any circumstances and at any age. According to Small Business Trends, the success rate of first entrepreneurship lies at 18% and the major reasons for failure are incompetence, unbalanced experience or lack of managerial experience, neglect, fraud, and lack of experiences related to goods or services (Mansfield, 2018). The economic benefits of the incubators should therefore not undermine the importance of senior entrepreneurship as an innovative way to tackle the economic inactivity of older adults. Moreover, experts underlined that costs of such incubators could be significantly lowered in the future if one model of support would be established, and further replicated in other locations and target groups without the initial costs of model development. And although similar social benefits could also be derived from other projects at lower costs (e.g., social inclusion projects), the innovative character of the senior entrepreneurial incubators cannot be underestimated, as the long term impact on the participants, as well as social and cultural outcomes for the society at large, are more difficult to quantify and measure and are perhaps still to be seen. Additionally, as stated by Khalil and Olafsen (2010): “Business incubators provide a proactive platform for early-stage entrepreneurial activities and trigger connectivity between different entrepreneurs, trainings and business advisory services. Regarding the interactions between like-minded incubates the value of a psychologically supportive environment cannot be overemphasized” (Khalil and Olafsen, 2010, p. 73).

Finally, attention should be drawn to the issue of qualitative measurement and evaluation of outcomes and impacts of social programs, such as senior entrepreneurship incubators. The assessment of the social impact of social innovations is a challenging task, as there is no coherent approach to the measurement of social effects. By their very nature, it is hard to measure social and environmental value due to the danger that such important benefits become subordinated to economic indicators that can claim greater rigor in terms of data quality (Arvidson et al., 2010). One of the approaches in measuring social impact is the Social Return on Investment (SROI) approach, which is still in its development stage rather than being an established methodological approach. SROI is described as an “approach toward identifying and appreciating value created. It involves reviewing the inputs, outputs, outcomes, and impacts made and experienced by stakeholders of an organization in relation to the activities of an organization, and putting a monetary value on the social, economic and environmental benefits and costs created by an organization” (ibid., 6). The approach is focused on attributing financial value to inputs and outputs, leading to the final process of calculating the SROI ratio. Other principles of SROI approach include: stakeholders' engagement, understanding the change, valuing what matters, or being transparent and the general approach should guarantee a very careful judgment of the social and/or environmental impacts. Arvidson et al. (2010) underline: “In order for a comprehensive and credible SROI assessment to take place, organizations will need access to evidence based on both quantitative and qualitative data, some of which is quantifiable and some of which is not.” The implementation of a qualitative approach to the evaluation of social projects is both challenging and necessary. In case of the senior entrepreneurship incubators, the quantitative outcomes and impacts were measures in all the cases but were at the same time criticized by the implementing organizations themselves as being too narrow. This approach stems from the requirements of the funding institutions, which demand hard quantitative data about the results of financing. The qualitative approach to the evaluation of results was less common and did not adhere to any specific pre-defined criteria and is also not among requirements for final reports. This qualitative approach to measuring outcomes would need to involve additional resources (e.g., for in-dept interviews with participants or other stakeholders) and be significantly extended in time (to allow for estimations of long-term impacts). Moreover, a for qualitative evaluation would be needed in order to reflect the SROI guidelines, such as to “measure what matters.” For a proper qualitative evaluation of impacts of the senior entrepreneurship incubators on the individual, communal, as well as organizational level it would be recommended to adapt an approach close to or similar to SROI approach, which simultaneously would require an investment in additional financial and personal resources, as well as methodological rigor of the qualitative evaluation.

## Ethics Statement

The individual interviews in the project were carried out in accordance with the ethical principles of good scientific practice of German Research Foundation (Deutsche Forschunsgemeinschaft). The approval of an Ethics Committee was not required for this study as per applicable institutional and national guidelines. Before each interview an explanation of the purpose of the study and the information about the audio data gathered during the interview was given to each participant. All the participants were asked for a permission to record the interviews and an oral informed consent was received from all of them.

## Author Contributions

The leading author of the paper is JS. The parts she prepared are: the general concept and outline of the publication, the introduction, the empirical part, as well as discussion and conclusions. AF and JM prepared the whole theoretical part, made comments, suggestions and corrections to the rest of the article.

### Conflict of Interest Statement

The authors declare that the research was conducted in the absence of any commercial or financial relationships that could be construed as a potential conflict of interest.

## Footnotes

^1^Project MOMENT—Making of Mature Entrepreneurs. Funded by the German Research Foundation (Deutsche Forschungsgemeinschaft); the implementation period: 01.2017-12.2019.

^2^The empirical data gathered in Germany did not allow for a similar analysis, as no specific incubators for senior entrepreneurship were identified in the project. The cases found in Poland reflect a very specific financing period (2007–2013) of the EU, where Poland was a major beneficiary of funds for social projects which allowed carrying out of these specific incubators.

^3^Until 2012 one could retire at the age of 65 or even earlier (for women). After 2013, the retirement age would be gradually increased until reaching the age of 67. Thus, men would reach the target retirement age of 67 in 2020, and women in 2040. The reform was later abolished by the government under the Prawo i Sprawiedliwość (PiS) Party in 2017, and the retirement age returned to 60 for women and 65 for men.

^4^The interviews with entrepreneurs are numbered as “ENTR 11,” “ENTR 12,” etc. The interviews with experts use “EXP” code and a number.

^5^The planned number of expert interviews in Poland is 20 and 30 for the entrepreneurs.

^6^Within the framework of Human Capital Operational Program Priority VI “labor market open for all,” measure 6.2 “Support and promotion of entrepreneurship and self-employment.”

^7^The verifiability of this method is the highest among all known recruitment methods, and its effectiveness is assessed at 43–65% (Gdanska Fundacja Przedsiebiorczości, 2014, p. 5).

^8^As part of the Human Capital Operational Program, Priority VI “Open market for everyone,” Measure 6.1. “Improvement of the access to employment and support for professional activity in the region,” Sub-measure 6.1.1. “Support for people without employment in the regional labor market.”

^9^The exact number of closures is not known due to the lack of systematic monitoring of the projects, but as some experts suggested, the closures were about 80% of the cases.

^10^The videos are still available at the https://www.voutube.com/watch?v=q7dwAz11rbE.
